# Overweight, underweight, and obesity among male long‐distance professional drivers in Iran

**DOI:** 10.1002/1348-9585.12114

**Published:** 2020-02-05

**Authors:** Siamak Pourabdian, Parastoo Golshiri, Mohsen Janghorbani

**Affiliations:** ^1^ Department of Occupational Health School of Public Health Isfahan University of Medical Sciences Isfahan Iran; ^2^ Department of Community Medicine Medical School Isfahan University of Medical Sciences Isfahan Iran; ^3^ Department of Epidemiology and Biostatistics Isfahan University of Medical Sciences Isfahan Iran

**Keywords:** drivers, Iran, obesity, overweight, prevalence, risk factors

## Abstract

**Objective:**

Long‐distance professional drivers, as an occupational group, are hypothesized to have a higher risk of overweight and obesity. The aim of this study was to estimate the prevalence and risk factors of overweight, underweight, and obesity in long‐distance professional drivers.

**Methods:**

A cross‐sectional study was conducted on 36 625 male long‐distance professional drivers age ≥20 years, from April 2013 to Sept. 2016. Drivers were interviewed and underwent clinical and laboratory examinations including measures of blood pressure (BP), blood tests, anthropometric data, and visual and hearing acuity. The mean (standard deviation [SD]) age of participants was 41.9 (10.2) years with a mean (SD) duration of a professional driving of 15.3 (9.6) years and mean (SD) body mass index (BMI) was 25.7 (4.0) kg/m^2^.

**Results:**

The prevalence of overweight and obesity was 39.1% (95% confidence interval (CI) 38.6, 39.6) and 10.8% (95% CI 10.5, 11.1), respectively. A total percentage of 2.7 (95% CI 2.5, 2.9) were underweight. A total percentage of 10.6 had BMI 30‐40 kg/m^2^ and 0.2% had BMI ≥40 kg/m^2^. Using a stepwise binary logistic regression model for overweight, underweight, and obesity, age had a significant independent relationship with underweight, overweight, and obesity. Duration of work, BP, fasting plasma glucose, triglyceride, and cholesterol had significant independent positive association and smoking had a negative association with overweight/obesity when other covariates were considered.

**Conclusions:**

These findings indicate that overweight and obesity are prevalent among long‐distance professional drivers in Iran and appears to be similar to the general population.

## 1 INTRODUCTION

Overweight/obesity is recognized as one of the world's priority health challenges. In 2016, the World Health Organization (WHO) estimated that globally about 39% of men aged 18 years and above were overweight and 11% were obese.^1^ Long‐distance professional drivers are exposed to circumstances that encourage weight gain and associated health problems. Considering the working environment of long‐distance professional drivers, it is expected that their weight would be more than the general population. It is hypothesized that overweight and obesity rates are higher among professional drivers than among general population and other occupational groups^2^, ^3^ because their working environment is characterized by numerous stress factors such as lack of physical activity due to working in a fixed position, unhealthy nutritional habits, irregular working hours, and irregular sleep habits, sitting for long periods, and prolonged stress. Some studies in different countries reported a relatively higher prevalence of overweight and obesity among professional drivers.^2^, ^4^, ^5^, ^6^, ^7^

Occupational health problems of long‐distance professional drivers have not received significant attention in developing countries and little is known about the exact prevalence of underweight, overweight, and obesity in professional drivers in Iran. In Iran, 700 000 professional drivers are employed on a full‐time basis, a cohort which constitutes a great proportion of the Iranian population.^8^ Considering the importance of overweight and obesity as a main risk factor for chronic diseases as well as its association with sleep problems that increase risk of accident,^9^ we aimed to conduct a study to shed some light on the prevalence of underweight, overweight, and obesity and its related risk factors in long‐distance professional drivers. The baseline data would be useful for future comparison of the secular trend and to provide data on the epidemiology of occupational obesity in Iran.

## 2 SUBJECTS AND METHODS

The Iranian long‐distance professional drivers study is a nationwide project focused on collecting data on the health status of the unselected group of professional drivers. It was initiated from April 2013 to December 2016 to draw attention to the health situation of professional drivers in Iran. All long‐distance professional drivers need to undergo periodic medical examinations defined by Iranian occupational health regulations.^10^ These periodic exams are administered by the Ministry of Road and Urban Development of the Islamic Republic of Iran and drivers need to carry a health card indicating that they have passed these tests all the time when driving.^10^ The sample in this study is a subset of the 700 000 long‐distance professional drivers for whom screening data were available and carried out in one of the authorized occupational health clinics in Isfahan, Iran. All long‐distance occupational drivers who pass through Isfahan city were offered general health screening at the study clinic and included. According to a national protocol, general health screening included measurements of height, weight, neck circumference, blood pressure (BP), visual, and hearing acuity. Clinical and laboratory evaluation was also completed during this visit. A total of 36 625 male long‐distance professional drivers included. Entry criteria included male drivers ≥20 years. A questionnaire about medical history was administered as an interview. Information was also collected from each driver on gender, age, blood group, and the number of years of the professional driving.

Resting BP was measured after subjects had been seated for 10 minutes by using a mercury sphygmomanometer and appropriately sized cuffs, using standard techniques. Fasting (12 hours) plasma glucose (FPG), cell blood counts, total cholesterol, and triglyceride were measured using standardized procedures. The cholesterol and triglyceride were assayed using enzymatic tests with commercially available kits (Pars Azmoon Inc, Iran). Plasma glucose was measured by the glucose‐peroxidase colorimetric enzymatic method. Low‐density lipoprotein (LDL) concentration was calculated using the Friedewald formula.^11^ The anthropometric measurements include height, neck circumference (to the nearest cm) and weight (at the nearest even decimal) were taken with subjects in light clothing without shoes. The hearing examination included pure‐tone air and bone conduction audiometry. Visual fitness evaluation was performed by either an optometrist or trained physician using the Snellen Chart and Vision Screener. If any further evaluation is needed, the drivers are referred to ophthalmologists. These are carrying on every other year for those under the age of 40 and annually after that.

### 2.1 Ethics statement

Ethical approval was obtained from the Isfahan University of Medical Sciences ethical committee, compliant with the Declaration of Helsinki. This was a retrospective study based on routine medical procedure, and additional written consent was not required. The data were processed and analyzed by authorized medical personnel only and was anonymized and de‐identified prior to analysis.

### 2.2 Definitions

Body mass index (BMI) is recognized as the measure of overall obesity. The criteria for underweight, desirable weight, overweight, and classes I, II, and III obesity used in this study were based on BMI (weight/height^2^ [kg/m^2^]) and were consistent with the definitions set forth by the WHO and the National Heart, Lung, and Blood Institute as follows: underweight <18.5, desirable weight 18.5‐24.9, overweight 25‐29.9, class I obesity 30‐34.9, class II obesity 35‐39.9, and class III obesity ≥40.^12^, ^13^

### 2.3 Analysis

Continuous and categorical variables are expressed as means with standard error of the mean (SEM) or 95% confidence intervals (CIs) and percentages, respectively, unless otherwise specified. Statistical methods used included the Student's *t* test, chi‐square test, one‐way analysis of variance followed by the one‐way post hoc Tukey test, and stepwise binary logistic regression. Spearman's rho partial correlation analyses adjusting for age were performed to determine the linear relationship between the blood parameters and BMI. Age‐adjusted means were calculated and compared using general linear models. To estimate the risk factors of underweight, overweight, and obesity, a stepwise binary logistic regression was carried out with the SPSS for Windows (SPSS Inc). Variable age, duration of driving, FPG, systolic BP, triglyceride, cholesterol, and smoking were entered in models as categorical. Adjustment for age was examined in separate models. Systolic and diastolic BP were not included simultaneously in regression analysis to avoid colinearity that these independent variables may have. All tests for statistical significance were two tailed, with the level of significance at *α* < .05.

## 3 RESULTS

### 3.1 Characteristics

The drivers had a mean (standard deviation [SD]) duration of professional driving 15.3 (9.6) years and mean (SD) age of 41.9 (10.2) years. Table 1 shows the differences in the distribution of age and age‐adjusted duration of work, systolic and diastolic BP, cholesterol, FPG, and height, weight, and neck circumference among drivers with desirable weight, underweight, overweight, and obesity. As expected, all of the variables increased with increasing BMI class, except height, and current smokers which decreased with increasing BMI class. Drivers in our sample were slightly overweight. The age‐adjusted mean (SEM) BMI was 25.7 (0.02) kg/m^2^. Of the 36 625 male long‐distance professional drivers, 14 320 were overweight, 3947 were obese, and 985 were underweight. Those with overweight and obesity were older and had higher age‐adjusted neck circumference, FPG, BP, triglyceride, and cholesterol than those with desirable weight. The underweights were younger and had lower age‐adjusted neck circumference, FPG, triglyceride, and cholesterol than those with desirable weight. Triglycerides ≥150 mg/dL were found in 60% and cholesterol ≥190 was found in 34.7% of drivers. Considering smoking habits, 3991 (10.9%) of participants were current smokers. Underweight drivers were more likely to have smoked. Most (64%) had been professional drivers for 10 years or more. A total percentage of 17.2 had been professional drivers for less than 5 years.

**Table 1:** Age‐adjusted characteristics of drivers by normal weight, underweight, overweight, and obesity

Variables	Age‐adjusted mean (SEM)			
	Underweight	Desirable weight	Overweight	Obese
Number (%)	985 (2.7)	17373 (47.4)	14320 (39.1)	3947 (10.8)
Age at registration (y)	38.1 (0.32)	41.0 (0.08)	42.9 (0.09)	42.9 (0.16)*
Duration of work (y)	15.2 (0.24)	15.1 (0.06)	15.4 (0.06)	15.8 (0.11)*
BMI (Kg/m^2^)	17.5 (0.06)	22.8 (0.01)	27.6 (0.02)	33.1 (0.03)*
Height (cm)	174.5 (0.19)	174.1 (0.04)	174.8 (0.05)	174.7 (0.09)*
Weight (Kg)	53.4 (0.24)	69.4 (0.06)	84.4 (0.06)	101.0 (0.12)*
Neck circumference (cm)	34.4 (0.07)	35.9 (0.02)	37.6 (0.02)	39.5 (0.03)*
Systolic BP (mmHg)	110.7 (0.35)	115.4 (0.08)	119.2 (0.09)	122.6 (0.17)*
Diastolic BP (mmHg)	71.0 (0.35)	74.2 (0.08)	76.2 (0.09)	77.6 (0.18)*
Fasting blood glucose (mg/dL)	91.1 (0.60)	92.8 (0.14)	95.7 (0.16)	98.2 (0.30)*
Triglyceride (mg/dL)	140.7 (2.23)	169.1 (0.53)	190.1 (0.58)	194.1 (1.11)*
Total cholesterol (mg/dL)	156.7 (1.12)	173.9 (0.27)	182.9 (0.29)	185.2 (0.56)*
HDL (mg/dL)	37.6 (0.50)	39.4 (0.12)	40.3 (0.14)	40.7 (0.26)*
LDL (mg/dL)	96.0 (2.48)	106.9 (0.60)	110.2 (0.68)	110.5 (1.27)*
	%	%	%	%
Triglyceride (mg/dL)				
<150	68.8	46.3	33.2	30.1
≥150	31.2	57.7	66.8	69.9*
Total cholesterol (mg/dL)				
<190	88.1	69.9	60.6	56.7
≥190	11.9	30.1	39.4	43.3*
Smoking				
Never smoker	81.5	87.9	90.7	90.6
Current smoker	18.5	12.1	9.3	9.4*
Blood group				
A+	27.1	28.2	28.1	29.3
A−	3.2	3.1	3.0	2.8
AB+	8.1	6.7	6.3	7.2
AB−	0.4	0.8	0.7	0.4
B+	20.5	21.7	21.9	21.8
B−	2.0	2.4	2.4	2.5
O+	34.1	33.5	33.9	32.3
O−	3.2	3.6	3.7	3.7

Note

Age‐adjusted means were calculated using general linear models. Category definitions are based on WHO and NHLBI cut‐offs.^11^, ^12^ Underweight <18.5, normal weight = 18.5 to <25, overweight = 25 to <30, obese ≥30.

Abbreviations: BP, blood pressure; BMI, body mass index; HDL, high‐density lipoprotein cholesterol; LDL, low‐density lipoprotein cholesterol; NHLBI, National Heart, Lung, and Blood Institute; WHO, World Health Organization.

* *P* < .001

### 3.2 Prevalence

Table 2 presents the prevalence of desirable weight, underweight, overweight, and classes I, II, and III obesity. A total percentage of 47.4 of the drivers had desirable weights. Nearly half of the drivers were overweight or obese (49.9%). Overall 39.1% (95% CI 38.6, 39.6) drivers were overweight and 10.8% (95% CI 10.5, 11.1) were obese. A total percentage of 2.7 (95% CI 2.5, 2.9) were underweight. 9.3% (95% CI 9.0, 9.6) had class I obesity (BMI 30‐35 kg/m^2^), 1.3% (95% CI 1.2, 1.4) had class II obesity (BMI 35‐40 kg/m^2^), and 0.2% (95% CI 0.16, 0.25) had class III obesity (BMI ≥40 kg/m^2^).

**Table 2:** Prevalence (%) of underweight, overweight, and classes I, II, and III obesity

Weight category^a^	Cases	Prevalence (%) (95% CI)
Underweight	985	2.7 (2.5, 2.9)
Desirable weight	17 373	47.4 (46.9, 47.9)
Overweight	14 320	39.1 (38.6, 39.6)
Class I obesity	3408	9.3 (9.0, 9.6)
Class II obesity	463	1.3 (1.2, 1.4)
Class III obesity	76	0.2 (0.16, 0.25)
Obesity (BMI ≥30)	3947	10.8 (10.5, 11.1)

Abbreviations: BMI, body mass index; CI, confidence interval; NHLBI, National Heart, Lung, and Blood Institute; WHO, World Health Organization.

a Category definitions are based on WHO and NHLBI cut‐offs (11, 12). Underweight = BMI <18.5 kg/m^2^, overweight = BMI 25‐29.9 kg/m^2^, Class I obesity = BMI 30‐34.9 kg/m^2^, Class II obesity = BMI 35‐39.9 kg/m^2^, and Class III obesity = BMI ≥40 kg/m^2^.

### 3.3 Risk factors

Age‐adjusted BMI was strongly correlated with weight (*r* = .92), neck circumference (*r* = .68), age (*r* = .12), systolic (*r* = .28), and diastolic BP (*r* = .23), cholesterol (*r* = .17), triglyceride (*r* = .22), hemoglobin (*r* = .13), hematocrite (*r* = .11), and FPG (*r* = .14), and negative correlation with daily cigarette consumption (*r* = −.10) (*P* < .001).

As expected, the prevalence of overweight and obesity increased and the prevalence of underweight decreased with age (*P* < .001). The age‐adjusted prevalence of overweight and obesity increased with duration of driving, and was present in 35.0% (95% CI 33.8, 36.2) and 9.2% (95% CI: 8.4, 9.9) of drivers with driving duration less than 5 years and in 43.2% (95% CI: 42.4, 44.0) and 12.2% (95% CI: 11.7, 12.8) of those with driving duration ≥15 years. After age adjustment, this difference was statistically significant (*P* < .001) (Table 3).

**Table 3:** Univariate analysis of age‐adjusted mean (standard error [SEM]) body mass index and prevalence (%) of underweight, desirable weight, overweight, and obesity in 36 625 male drivers according to selected characteristics, Iran

Variables	Mean (SE)	Weight category^a^					
		Underweight (%)	Age‐adjusted OR (95% CI)	Overweight (%)	Age‐adjusted OR (95% CI)	Obesity (%)	Age‐adjusted OR (95% CI)
Age at registration (y)							
<30	27.7 (0.04)	5.4	1.00	28.7	1.00	7.9	1.00
30‐39	35.2 (0.03)	2.6	0.59 (0.50, 0.69)***	38.2	1.59 (1.48, 1.71)***	10.7	1.62 (1.45, 1.83)***
40‐49	45.2 (0.03)	2.1	0.52 (0.44, 0.62)***	42.6	1.98 (1.85, 2.13)***	11.8	2.01 (1.79, 2.26)***
50‐59	55.0 (0.03)	1.7	0.42 (0.39, 0.52)***	42.8	1.97 (1.82, 2.12)***	11.5	1.94 (1.71, 2.19)***
60	63.7 (0.08)	1.9	0.46 (0.29, 0.72)**	42.0	1.87 (1.63, 2.16)***	10.7	1.74 (1.39, 2.18)***
Duration of work (y)							
<5	5.0 (0.06)	3.4	1.00	35.0	1.00	9.2	1.00
5‐9	9.6 (0.05)	2.9	0.91 (0.74, 1.12)	37.4	1.12 (1.04, 1.21)**	9.6	1.10 (0.97, 1.24)
10‐15	13.4 (0.05)	2.3	0.87 (0.70, 1.07)	39.4	1.20(1.12,1.30)***	11.0	1.31 (1.16,1.48)***
15	22.9 (0.04)	2.0	1.12 (0.88, 1.41)	43.2	1.33 (1.23, 1.44)***	12.2	1.53 (1.35, 1.73)***
Fasting plasma glucose (mg/dL)							
<100	87.2 (0.07)	3.1	1.00	37.1	1.00	9.6	1.00
100‐125	109.2 (0.12)	1.6	0.74 (0.61, 0.89)**	44.2	1.38 (1.31, 1.46)***	13.7	1.69 (1.56, 1.83)***
≥126	180.9 (0.38)	0.3	0.20 (0.05, 0.82)*	51.2	2.09 (1.76, 2.47)***	20.4	3.35 (2.71, 4.14)***
Smoking (%)							
Never smoker	89.1	81.5	1.00	90.7	1.00	90.6	1.00
Current smoker	10.9	18.5	1.78 (1.50, 2.10)***	9.3	0.73 (0.68, 0.79)***	9.4	0.73 (0.65, 0.82)***
Systolic BP (mmHg)							
<140	116.5 (0.05)	2.7	1.00	38.8	1.00	10.4	1.00
≥140	150.9 (0.29)	0.9	0.80 (0.42, 1.51)	48.3	1.72 (1.49, 1.98)***	22.5	3.14 (2.64, 3.73)***
Diastolic BP (mmHg)							
<90	75.1 (0.04)	2.8	1.00	38.7	1.00	10.7	1.00
≥90	96.4 (0.68)	1.0	0.86 (0.12, 6.34)	40.2	1.56 (0.96, 2.54)	32.4	4.61 (2.76, 7.70)***
Triglyceride (mg/dL)							
<150	126.6 (0.46)	4.6	1.00	32.4	1.00	8.1	1.00
≥150	214.2 (0.38)	1.4	0.42 (0.36, 0.48)***	43.6	1.68 (1.60, 1.76)***	12.6	1.94 (1.80, 2.09)***
Cholesterol (mg/dL)							
<190	157.9 (0.14)	3.6	1.00	36.3	1.00	9.4	1.00
≥190	216.4 (0.20)	0.9	0.34 (0.28, 0.42)***	44.4	1.44 (1.37, 1.51)***	13.5	1.69 (1.58, 1.82)***

Note

Odds ratio (with 95% CI) calculated by binary logistic regression. Age‐adjusted means were calculated using general linear models.

Abbreviations: BP, blood pressure; BMI, body mass index; CI, confidence interval; NHLBI, National Heart, Lung, and Blood Institute; WHO, World Health Organization.

a Category definitions are based on WHO and NHLBI cut‐offs.^11^, ^12^ Underweight = BMI <18.5 kg/m^2^, overweight = BMI 25‐29.9 kg/m^2^, obesity = BMI ≥30 kg/m^2^.

* *P* < .5;

** *P* < .01;

*** *P* < .001.

To determine the independent associations of the prevalence of underweight, overweight, and obesity, a forward stepwise binary logistic regression was performed to test 7 variables: age, duration of driving, systolic BP, FPG, cholesterol, triglyceride, and smoking. Underweight drivers were more likely than those of desirable weight to be younger, to smoke, to have lower FPG, cholesterol, and triglyceride. Overweight and obese drivers were more likely than those of desirable weight to have a higher duration of professional driving, FPG, systolic BP, triglyceride, and cholesterol. Desirable weight drivers were more likely than those of overweight/obesity to be a smoker. As expected there was a statistically significant interaction between age and duration of driving (Table 4).

**Table 4:** Multivariate analysis of factors related to prevalence of underweight (BMI <18.5), overweight (BMI 25‐29.9), and obesity (BMI ≥30) (stepwise binary logistic regression model), significant adjusted odds ratios (95% CI)

Variables	Underweight	Overweight	Obesity
Age at registration (y)			
<30	1.00	1.00	1.00
30‐39	0.69 (0.57, 0.84)***	1.40 (1.29, 1.52)***	1.35 (1.18, 1.55)***
40‐49	0.69 (0.57, 0.85)***	1.50 (1.37, 1.64)***	1.28 (1.10, 1.49)**
50‐59	0.60 (0.48, 0.77)***	1.32 (1.20, 1.46)***	1.01 (0.85, 1.19)
60	0.72 (0.45, 1.14)	1.17 (0.99, 1.37)	0.77 (0.59, 1.00)*
Duration of work (y)			
<5	1.00	1.00	1.00
5‐9	—	1.07 (0.98, 1.15)	1.02 (0.90, 1.16)
10‐15	—	1.09 (1.004, 1.18)*	1.18 (1.04, 1.34)*
15	—	1.22 (1.13, 1.32)***	1.37 (1.21, 1.55)***
Fasting plasma glucose (mg/dL)			
<100	1.00	1.00	1.00
100‐125	0.81 (0.67, 0.98)*	1.33 (1.26, 1.41)***	1.55 (1.43, 1.69)***
≥126	0.23 (0.06, 0.93)*	1.99 (1.67, 2.37)***	3.07 (2.47, 3.83)***
Smoking			
Non‐smokers	1.0	1.00	1.00
Current smokers	1.90 (1.58, 2.28)***	0.69 (0.64, 0.75)***	0.68 (0.60, 0.77)***
Systolic BP (mmHg)			
<140	1.00	1.00	1.00
≥140	—	1.81 (1.56, 2.09)***	3.10 (2.58, 3.71)***
Triglyceride (mg/dL)			
<150	1.00	1.00	1.00
≥150	0.49 (0.42, 0.57)***	1.51 (1.44, 1.59)***	1.64 (1.51, 1.78)***
Cholesterol (mg/dL)			
<190	1.0	1.00	1.00
≥190	0.46 (0.37, 0.57)***	1.19 (1.13, 1.26)***	1.33 (1.23, 1.44)***

Abbreviations: BMI, body mass index; BP, blood pressure; CI, confidence interval.

* *P* < .05;

** *P* < .01;

*** *P* < .00.

## 4 DISCUSSION

In this study of 36 625 male long‐distance professional drivers, we found an overall prevalence of overweight and obesity of 39.1% and 10.8%. In contrast, underweight has a low prevalence (2.7% of drivers present BMI values lower than 18.5). The results of this study demonstrate that overweight and obesity among the long‐distance professional drivers in Iran appear to be similar to the same age and gender group in the general population, as about half of the long‐distance professional drivers (49.9%) was characterized as overweight or obese. According to the WHO report, about 50% of the Iranian adult population are overweight or obese.^14^ In 2016, 39% of Iranian men aged >18 years were overweight and about 11% were obese.^15^ The prevalence of underweight among men in Iran is 6.3%.^16^ Similar to this study, the mean BMI for Iranian men is 25.3 kg/m^2^.^17^ A few studies have investigated overweight and obesity or its related components in long‐distance professional drivers. The prevalence of overweight and obesity among Brazilian truck drivers was 47.8% and 16.2%^4^ and in an Israeli study was 37.5% and 15%.^5^ Another Brazilian study also reported a high prevalence rate of overweight and obesity: 57.5% of the population of bus drivers was overweight and approximately 19.5% was obese.^18^ In an Italian study 61% of professional drivers were either overweight or obese.^19^ In one study carried out in Poland, this rate was 62%^20^ and in another study in Mexico, it was reported to be 75.2%.^21^ In the United States, the prevalence of obesity among male employees who work as motor vehicle operators was found to be 31.7%^6^ and if we consider the data from a national survey conducted in 2010 among the US long‐haul truck drivers, 69% were obese and 17% drivers were morbidly obese (BMI >40 kg/m^2^).^2^ The prevalence of overweight and obesity in long‐distance professional drivers in Iran is lower than the values reported in Brazil,^4^ Israel,^5^ Italy,^19^ Poland,^20^ and India, showed 43.3% of drivers were overweight and 22.2% were obese,^22^ and specially the United States.^2^, ^6^ Estimates of the prevalence of overweight and obesity will depend upon methodological factors, the definition of obesity used, and the composition of the community examined by age, gender, ethnicity, and social class, making comparisons between studies of limited values.

The prevalence and pattern of obesity vary substantially from nation to nation,^14^ and its current prevalence (BMI ≥30) among the general population ranges from as low as <2% in certain Asian and African nations (Ethiopia, Bangladesh, Nepal, Eritrea, and Madagascar) to as high as >61% in Nauru.^15^

Considering the working environment of long‐distance professional drivers (lack of access to physical activity due to working in a fixed position and unhealthy eating, irregular working hours, and irregular sleep habits, sitting for long periods, and prolonged stress), it is expected that their weight would be more than the general population. Several studies in different countries also reported a relatively high prevalence of overweight and obesity among professional drivers.^2^, ^4^, ^5^, ^6^, ^18^, ^19^, ^20^ However, in our study professional drivers were men, younger, and assumed to be more active than general population. The reason for the observed difference in overweight and obesity can be claimed to be the difference in the age, gender, ethnicity, nutritional habits, and smoking. Iranian professional drivers are heavy smokers and seldom drink alcohol due to religious believes. The reasons for less risk of overweight/obesity in professional drivers in Iran need to be further studied.

Univariate analysis (Table 3) shows an expected pattern of association for many variables with underweight, overweight, and obesity. In multivariate analysis fewer remains independently associated.

Duration of driving was associated with the prevalence of overweight and obesity. When adjusting for other risk factors by multivariate analysis, duration of driving was a significant risk factor for both overweight and obesity. But there was statistically significant interaction between age and duration of driving and effects of both variables have not been independent. This association was also seen in a Colombian^19^ and Italian^7^ study that found a positive association between time spent traveling in motor vehicles per week and overweight and abdominal obesity.

Consistent with prior studies in the general population,^23^, ^24^, ^25^, ^26^ the present study found similarly increasing the prevalence of overweight and obesity with increasing age in a long‐distance professional driver population.

In univariate analysis, blood glucose was associated with a significant increase in the risk. After age adjustment, the level of hyperglycemia, as measured by one fasting blood glucose, had a positive association with overweight and obesity and negative association with underweight. After adjustment for other covariates in the multivariate analysis, the level of fasting glucose was significant.

The effect of BP on the risk of overweight and obesity is an important but difficult issue because BP rises as BMI and age increase.

A high proportion of the drivers in this study smoked, and smoking was inversely related to overweight/obesity. The negative association between smoking and overweight and obesity might be partly due to its effects on the metabolic rate, energy intake and storage, and energy expenditure.^27^, ^28^ Although a greater risk of excess weight is found among non‐smokers, many studies have shown that smoking has a larger impact on morbidity and mortality than any small increase in BMI.^29^, ^30^

Another finding that requires further elaboration is the high prevalence of dyslipidemia in male long‐distance professional drivers. Conflicting results have been observed in different studies. In Sweden, 440 professional bus and truck drivers were compared with a control group of 1000 subjects. It did not show differences in total cholesterol, HDL‐C, and triglycerides.^31^ On the other hand, outcomes of the study conducted in Serbia showed that 79% of the drivers suffered from dyslipidemia^32^ and 35.7% of bus drivers in Brazil were found to have hypercholesterolemia.^18^ The study focused on long‐haul truck drivers in the United States showed that 22% were taking medicine for or had been told they had high cholesterol.^2^ A study in Poland showed that in a non‐selected group of European professional drivers, lipid abnormalities, and excess weight are highly prevalent.^33^ A total percentage of 72.2 of the studied population had LDL level >115 mg/dL, and abnormally low levels of HDL‐C were found in 84.4% of drivers. Sixty percentage of our study drivers had triglycerides ≥150 mg/dL and cholesterol ≥190 was found in 34.7% of drivers.

Our study has several strengths and limitations. The strengths of this study include the large sample size and detailed information on potential confounding factors such as demographics, anthropometric, BP, and biochemical factors. Our sample should also be reasonably representative of Iranian long‐distance professional drivers. As a cross‐sectional study, the present analysis is limited in its ability to elucidate causal relationships between risk factors and underweight, overweight, and obesity. Because there was no information about health behaviors of drivers we cannot evaluate whether the increased overweight/obesity risk was mediated by health behaviors, such as physical activity and nutrition status. In addition, as the study participants were white men, the results may not apply to other racial/ethnic groups, or to women.

Despite the above limitations, the findings here add to our understanding of the epidemiology of underweight, overweight, and obesity in long‐distance professional drives in Iran.

In summary, overweight and obesity are prevalent in Iranian long‐distance professional drivers and overweight, and obesity in long‐distance professional drivers appears to be similar to the general population. With an estimated prevalence of about 50%, overweight and obesity clearly pose a formidable health threat to Iranian long‐distance professional drivers, which need more programs of health promotion and lifestyle changes. Further efforts are needed to reduce the burden of underweight, overweight, and obesity in professional drivers.

## DISCLOSURE

*Approval of the research protocol*: The study was approved by the Isfahan University of Medical Sciences ethics committee, and was conducted in accordance with Good Clinical practice. *Informed consent*: This was a retrospective study based on routine medical procedure, and additional written consent was not required. The data were processed and analyzed by authorized medical personnel only and was anonymized and de‐identified prior to analysis. *Registry and the registration no. of the study/trial*: N/A. *Animal studies*: N/A. *Conflicts of interest*: The authors declare that they have no conflicts of interest concerning this article.

## AUTHOR CONTRIBUTIONS

Janghorbani M conceived and designed the study, analyzed and interpreted the data, and drafted the manuscript; Pourabdian S recruited samples and contributed to the discussion and revision of the manuscript. Golshiri P contributed to the discussion and revision of the manuscript. All authors approved the final version submitted for publication.

## Data Availability

We would like to inform you that all the datasets and/or analyzed during this study are available from the corresponding author on reasonable request.
